# BK Polyomavirus bkv-miR-B1-5p: A Stable Micro-RNA to Monitor Active Viral Replication after Kidney Transplantation

**DOI:** 10.3390/ijms23137240

**Published:** 2022-06-29

**Authors:** Baptiste Demey, Marine Bentz, Véronique Descamps, Virginie Morel, Catherine Francois, Sandrine Castelain, Francois Helle, Etienne Brochot

**Affiliations:** 1 Laboratoire de Virologie, Centre Hospitalier Universitaire, F-80000 Amiens, France; descamps.veronique@chu-amiens.fr (V.D.); morel.virginie@chu-amiens.fr (V.M.); francois.catherine@chu-amiens.fr (C.F.); castelain.sandrine@chu-amiens.fr (S.C.); 2 UR UPJV 4294, Agents Infectieux, Résistance et Chimiothérapie (AGIR), Centre Universitaire de Recherche en Santé, Université de Picardie Jules Verne, F-80000 Amiens, France; marine.bentz@u-picardie.fr (M.B.); francois.helle@u-picardie.fr (F.H.)

**Keywords:** BKPyV, kidney transplantation, micro-RNA, miRNA, BK polyomavirus, biomarker, stability, exosomes

## Abstract

Background: Bkv-miR-B1-5p is a viral micro-RNA (miRNA) specifically produced during BK polyomavirus (BKPyV) replication. Recent studies have suggested using bkv-miR-B1-5p as a biomarker to monitor viral infection and predict complications in kidney transplant patients. To identify the technical limitations of this miRNA quantification in biological samples, knowledge of its stability and distribution in the extracellular compartment is necessary. Moreover, a proof of concept for using bkv-miR-B1-5p as a biomarker of active replication in chronic infection is still missing in the published literature. Methods: The stability of bkv-miR-B1-5p was evaluated in samples derived from cell cultures and in urine from BKPyV-infected kidney transplant recipients. The miRNA was quantified in different fractions of the extracellular compartment, including exosomes, and protein binding was evaluated. Finally, we developed an in vitro model for chronic culture of BKPyV clinical isolates to observe changes in the bkv-miR-B1-5p level during persistent infections. Results: Bkv-miR-B1-5p is a stable biomarker in samples from humans and in vitro experiments. Marginally associated with the exosomes, most of the circulating bkv-miR-B1-5p is bound to proteins, especially Ago2, so the miRNA quantification does not require specific exosome isolation. The bkv-miR-B1-5p level is predictable of viral infectivity, which makes it a potential specific biomarker of active BKPyV replication after kidney transplantation.

## 1. Introduction

The BK Polyomavirus (BKPyV), a member of the *Polyomaviridae* family, is a common virus in the general population [1]. BKPyV infectious viral particles are non-enveloped, with a diameter of ~45 nm. The capsid which encloses the genome is mainly composed of pentamers of viral protein VP1 associated with viral proteins VP2 and VP3 in the inner part. Genetic variation of the VP1 sequence divides BKPyV into four major subtypes (I, II, III and IV). Subtype I, the most prevalent, is distributed worldwide, whereas the others are less frequently found, with geographic heterogeneity [2]. The seroprevalence of this strictly human virus is close to 100% at the age of 10 years and then decreases to 70–80% in the elderly [3]. The primary infection is known to occur in childhood, causing mild symptoms or no symptoms at all. Then, a slight infection persists in various tissues and cells for which the virions have high tropism, especially in the urinary tract. In the immunocompetent population, this life-long infection is harmless and simply marked by periodical shedding in urine [2]. In contrast, in immunocompromised patients, the viral replication can go haywire, leading to significant clinical outcomes. Hemorrhagic cystitis is the main manifestation in hematopoietic stem cell recipients or patients under chemotherapy such as cyclophosphamide [4]. An intense replication in kidney transplant recipients (KTRs) affects the graft integrity, causing decreased renal function [5]. The final stage of the disease is BKPyV nephropathy (BKPyVN), which leads to graft loss [6].

To date, there is no specific approved therapy to prevent or treat BKPyV infection [7]. Fluoroquinolones, in particular ciprofloxacin, have been evaluated for prophylaxis but the clinical benefit was not sufficient [8,9]. Intravenous immunoglobulin solutions, which contain BKPyV neutralizing antibodies, have not shown a clear efficiency for prevention or treatment of BKPyV infection [10]. Cidofovir and leflunomide, despite numerous studies, did not prove clinical improvement and caused side effects (nephrotoxicity, hepatotoxicity, myelotoxicity) [11,12,13]. The only form of therapeutic management is the lightening of the immunosuppressive regimen, at the risk of promoting immunological complications [14,15,16].

Worldwide, almost 79,000 patients received a kidney transplant in 2020 [17]. Up to 40% of KTRs are concerned by BKPyV outcomes, but typical signs of infection are absent or discrete at first [18]. Given that the risk factors are not clearly defined [19], preemptive monitoring of the BKPyV replication is recommended in all KTRs [18]. BKPyV DNA quantification in urine and plasma has become the gold standard diagnostic strategy. In fact, this BKPyV periodic screening method statistically reduced the BKPyVN prevalence in studied populations [20]. Retrospective analyses showed that a BKPyV DNAuria superior to 10^7^ IU/mL can predict DNAemia [21]. Moreover, there is an important risk of BKPyVN if DNAemia exceeds 10^4^ IU/mL [18]. The immunosuppressive regimen should be reduced once the BKPyV replication is diagnosed, but actual recommendations do not define criteria to reset to normal doses. As BKPyV DNA can be detected for months in urine and plasma, we lack a specific marker that would indicate the cessation of active viral replication and a return to a persistent status. If so, nephrologists would be able to adapt the therapeutics as soon as possible and thus limit the risk of immunological complications such as acute rejection.

BKPyV is a virus with circular double-stranded DNA that replicates in two directions from a unique origin inserted in the non-coding control region (NCCR) [2]. The viral genome encodes sequentially for early proteins that initiate viral replication, then for late proteins implicated in the structural assembly of virions. Early genes encode for the large T, small T, and truncated T antigens (TAg, tAg, and truncTAg, respectively). Late genes encode for the VP1, VP2, VP3, and Agno proteins. Transcription in the direction of late genes is also responsible for the generation of a pre-miRNA whose sequence is perfectly complementary to the TAg sequence [22]. Recently, it was shown that the RNA polymerase II could circle the genome multiple times, producing transcripts with tandem repeats of BKPyV sequences [23,24]. This phenomenon places the pre-miRNA sequence in a genome-sized intron and promotes a higher miRNA expression level. A typical mechanism of maturation involving Dicer and Drosha activity leads to the generation of two mature miRNAs: bkv-miR-B1-3p and bkv-miR-B1-5p. The bkv-miR-B1-3p sequence is identical to the JC polyomavirus (JCPyV) 3p miRNA, but the bkv-miR-B1-5p is specific to the BKPyV, with a highly conserved sequence [25,26]. It was shown that bkv-miR-B1-3p is more expressed than bkv-miR-B1-5p during BKPyV infection [27].

Since bkv-miR-B1-5p is specific to the BKPyV and only produced during active replication of the viral genome, its suitability as a novel biomarker for monitoring BKPyV infection can be evaluated. We previously hypothesized that bkv-miR-B1-5p quantification of KTRs in urine would be a relevant non-invasive strategy to monitor BKPyV replication once infection is diagnosed using DNA quantification [28]. However, the sensitivity of the method is still limited and knowledge of bkv-miR-B1-5p properties in extracellular environments is required to improve the assays and to better interpret the results. Many studies have focused on BKPyV miRNA detection in urine, plasma, or cerebrospinal fluid [29,30,31]. The strategies for the purification of miRNAs were heterogeneous, with some authors preferring extraction of the exosomal fraction while others chose to analyze untreated total samples. Furthermore, most of the articles about BKPyV miRNAs are based on retrospective experiments using samples that were frozen for a long time. Nevertheless, none of the studies assessed the stability of BKPyV miRNAs following freezing or the room temperature conservation of the samples. In addition, we know neither the part of exosomal bkv-miR-B1-5p nor the degree of protein binding for the remaining part. Generally, a small quantity of miRNA is included in exosomes, especially those produced from viral genomes [32,33]. Most of the circulating miRNAs are usually bound to the Argonaute protein family, whose most dominant member is Argonaute2 (Ago2), in order to confer great extracellular stability [34,35,36,37]. These characteristics have never been experimentally demonstrated for bkv-miR-B1-5p.

In the present study, we evaluated the stability of bkv-miR-B1-5p after freezing or room temperature storage, in infected cultured cells and fresh urine of KTRs suffering from BKPyV active replication. We analyzed the part of circulating bkv-miR-B1-5p that is contained in exosomes and bound to the Ago2 protein to define which strategy would be better for bkv-miR-B1-5p purification from biological fluids. Finally, using an in vitro model of renal proximal tubular epithelial human cell infection with BKPyV clinical isolates, we showed that bkv-miR-B1-5p levels are related to the replication intensity.

## 2. Results

### 2.1. Stability of In Vitro Generated bkv-miR-B1-5p

The release of bkv-miR-B1-5p in conditioned media from cell culture infected by the Dunlop BKPyV strain [38], the most commonly used strain for in vitro studies, could involve several mechanisms. We supposed that the two main ways for bkv-miR-B1-5p to exit the cell were inclusion in exosomes or discharge after cell lysis caused by BKPyV infection. Depending on the process, the extracellular stability of the miRNA could be altered as lytic infection could imply more protein binding and higher stability, whereas passive excretion through exosomes could reduce the bkv-miR-B1-5p stability because of the limited exosome lifetime. 

**Figure 1:**
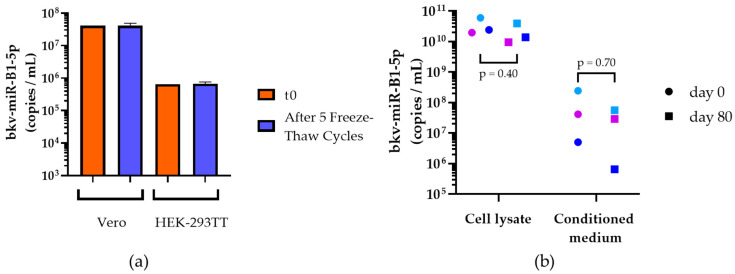
Bkv-miR-B1-5p quantification in samples from in vitro models. (**a**) bkv-miR-B1-5p level in conditioned medium from Vero cells (left) or HEK-293TT cells (right) at the time of collection (orange) or after five freeze–thaw cycles (blue). Colored bars represent mean, and error bars represent the standard deviation of three independent experiments; (**b**) bkv-miR-B1-5p level in cell lysate (left) and conditioned medium (right) from Vero cells infected with BKPyV. Dots correspond to the initial quantities and squares correspond to the quantities measured after 80 days storage at room temperature. Pairs of samples are color matched. *p* values were obtained by a Mann–Whitney test.

First, monkey-derived Vero cells and human embryonic kidney HEK-293TT cells were infected with BKPyV. Three days later, conditioned media were collected and bkv-miR-B1-5p was quantified either in untreated media or after five freeze–thaw cycles from −20 °C to room temperature. For both cell lines, the bkv-miR-B1-5p level did not differ after freeze–thaw cycles compared to untreated samples (Figure 1a). The bkv-miR-B1-5p level in conditioned media from Vero cells was higher than that from HEK-293TT cells. Thus, this result confirms that there was no bias in bkv-miR-B1-5p quantification in the retrospective study, even if the templates were thawed multiple times. 

Then, we compared the degradation of bkv-miR-B1-5p in the conditioned medium and lysate of Vero infected cells when the samples were kept for 80 days at room temperature, compared to quantities at the time of collection. A previous article on cellular human miRNA showed that it was stable for 60 days in lysed cell solution while the miRNA level decreased, importantly, if the conditioned medium was kept this long at room temperature. Interestingly, we highlighted an equivalent bkv-miR-B1-5p stability at day 80 in the conditioned medium and in the cell lysate of infected Vero cells, as shown in Figure 1b. In fact, 80-day room temperature conservation had no statistically significant effect on bkv-miR-B1-5p measures in the cell lysate or the conditioned medium (*p* = 0.40 and *p* = 0.7025, respectively, with the Mann–Whitney test). Moreover, the bkv-miR-B1-5p level was much higher in cell lysate as compared to conditioned media. Taken together, this suggests that BKPyV miRNAs are highly stable in extracellular media and cell lysate even after prolonged storage at room temperature. Contrary to previous studies on cellular miRNA stability [34], we showed that bkv-miR-B1-5p remained measurable without significant changes after extreme storage conditions, probably because of the cytopathic effect of the infection exacerbating lytic release of miRNAs.

### 2.2. Stability of bkv-miR-B1-5p in Human Urine

**Figure 2:**
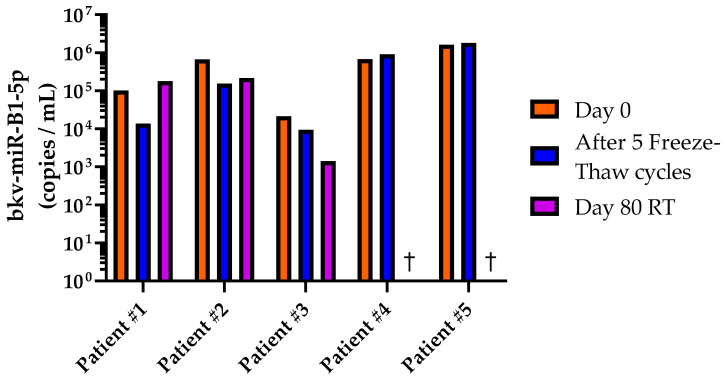
bkv-miR-B1-5p levels in urine from patients diagnosed with a BKPyV infection. The measurements were performed at day 0 (orange bars), after five freeze-thaw cycles following 80 days of −20 °C conservation (blue bars), or after 80 days of room temperature (RT) conservation (purple bars). †: zero value. The five experiments were independently performed between March 2021 and December 2021.

As bkv-miR-B1-5p has the potential to expand the diversity of BKPyV biomarkers in clinical practice, the medical community needs to evaluate the accuracy of the assays and their impact on therapeutic management. The first step would be retrospective analysis of a large cohort of patients. Hence, knowledge about bkv-miR-B1-5p stability in human samples is crucial. Five fresh urine samples from distinct patients with a diagnosed BKPyV infection were collected. Biological and clinical information about the patients are described in Table S1. The bkv-miR-B1-5p level was measured at day 0 in untreated urine. An aliquot was set aside at room temperature for 80 days. Another aliquot was frozen and conserved at −20 °C for 80 days. At day 80, bkv-miR-B1-5p was quantified in the room temperature urine and in the −20 °C urine, after 5 freeze-thaw cycles. As shown in Figure 2, bkv-miR-B1-5p was still detectable with similar levels between day 0 and after the freeze–thaw cycles for all the patients. The change in miRNA load was always less than 1 log copies/mL and a decrease of more than 0.5 log/mL was only observed for patients #1 and #2 (−0.87 and −0.63 log/mL, respectively). According to our in vitro results, bkv-miR-B1-5p was still highly detectable in urine from patients #1, #2, and #3 after 80 days at room temperature. Nevertheless, bkv-miR-B1-5p was not quantifiable in urine anymore from patients #4 and #5 without knowing if it was due to a degradation of the molecules or to the sensitivity limit of the assay. Overall, the Mann–Whitney test did not reveal significant differences between the bkv-miR-B1-5p level at day 0 and after five freeze–thaw cycles (*p* = 0.841), or after 80 days of room temperature storage (*p* = 0.097). Therefore, bkv-miR-B1-5p appears to be a relevant non-invasive biomarker with high stability in frozen human urine, but the level measures must be carefully interpreted if the samples are not properly conserved.

### 2.3. Part of Exosomal bkv-miR-B1-5p in Extracellular Compartment

**Figure 3:**
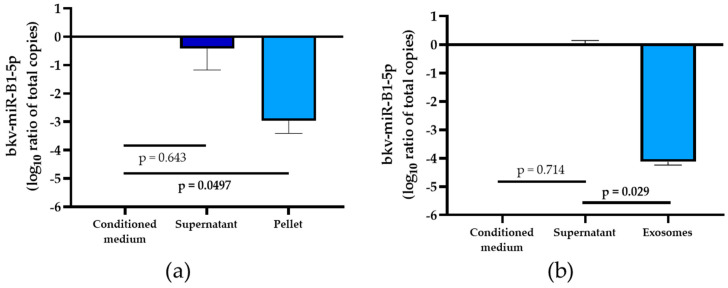
Log10 ratio of total bkv-miR-B1-5p amounts in the extracellular environment of BKPyV-infected Vero cells. Colored bars represent mean, and error bars represent the standard deviation of 3 independent experiments. *p* values were obtained by a Mann–Whitney test for both (**a**) and (**b**) experiments. (**a**) Log10 ratio of total bkv-miR-B1-5p amount in conditioned medium, supernatant and pellet resulting from a 100,000× *g* ultracentrifugation for 2 h at 4 °C; (**b**) Log10 ratio of total bkv-miR-B1-5p amount in conditioned medium, supernatant and exosome eluate resulting from µMACS Exosome Isolation Kit Pan, human (Miltenyi^®^).

Now that the stability of bkv-miR-B1-5p in the extracellular medium was described, we sought to evaluate the proportion of viral miRNA contained in exosomes compared to the rest of the extracellular compartment in order to optimize the purification strategy. Thus, conditioned medium was collected from Vero cells infected with BKPyV (Multiplicity Of Infection: MOI = 1). The liquid was processed by a typical differential centrifugation method to purify exosomes [34]. The bkv-miR-B1-5p level did not decrease after centrifugation at 10,000× *g* for 10 min and subsequent filtration through 0.22 μm filters (data not shown). These results are in favor of an absence of association of the miRNA with cellular debris and microvesicles. Ultracentrifuging the samples at 100,000× *g* for 2 h permitted the collection of an exosome-free supernatant. The ultracentrifugation pellet was then washed carefully with PBS and the solution was centrifuged at 100,000× *g* for 2 h again. The resulting pellet was lysed in Qiazol and the total amount of bkv-miR-B1-5p contained in the exosomes was quantified. There was no statistically significant change in the bkv-miR-B1-5p total amount of the ultracentrifugation supernatant compared to the initial conditioned medium (*p* = 0.643, Figure 3a). In contrast, the mean exosomal fraction of bkv-miR-B1-5p was 2.97 log10 less important than in the total conditioned medium (*p* = 0.0497). This first experiment thus highlighted that only 1‰ of extracellular bkv-miR-B1-5p is included in exosomes. 

To further explore this repartition, we used a specific exosome isolation assay based on immunocapture technology in order to isolate the exosomes from a 30X concentrate after 10 kDa molecular weight cutoff (MWCO) ultrafiltration of the conditioned medium. Like the previous observations, we observed a very low proportion of extracellular bkv-miR-B1-5p in exosomes (Figure 3b). In fact, we observed a 4.14 log10 difference in the quantity of this specific BKPyV miRNA in the exosome eluate as compared to the total amount of bkv-miR-B1-5p in the remaining supernatant, which was statistically significant (*p* = 0.029).

These results prove that only a few bkv-miR-B1-5p are found in exosomes and suggest that bkv-miR-B1-5p must be searched in total native samples for higher testing sensitivity, if the exosomal fraction is not specifically targeted.

### 2.4. Association of bkv-miR-B1-5p with Ago2 Protein

**Figure 4:**
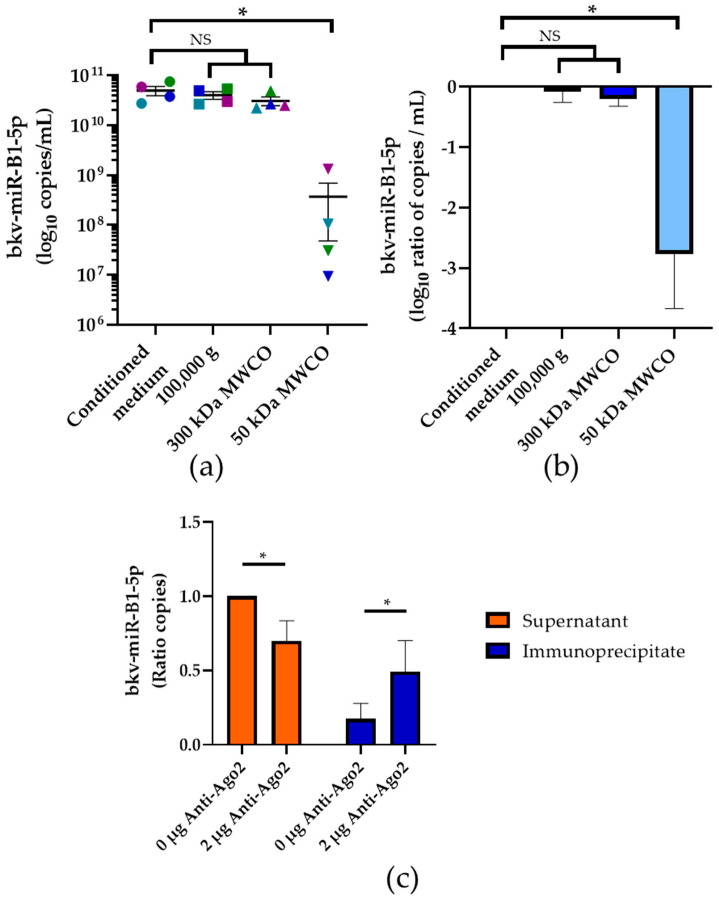
bkv-miR-B1-5p level (**a**) and log10 ratio (**b**) in conditioned medium from BKPyV-infected Vero cells, supernatants obtained after centrifugation at 100,000× *g* for 2 h at 4 °C, and filtrates obtained after filtration through 300 kDa and 50 kDa MWCO membranes. Lines (**a**) and colored bars (**b**) represent mean, and error bars represent the standard deviation of four independent experiments. Paired samples are color-matched; (**c**) Ratio of bkv-miR-B1-5p amount in anti-Ago2 and control supernatant (orange) and immunoprecipitate (blue). Colored bars represent mean and error bars represent the standard deviation of 4 independent experiments. Pairs of samples are color-matched. *p* values were obtained by a Mann-Whitney test (*: *p* < 0.05).

As we showed that most of bkv-miR-B1-5p circulates in the extracellular environment independently of exosomes, the stabilization of the miRNA outside the cell remained unclear. Ago2, a 96 kDa protein, is known to carry circulating miRNA in mammalian extracellular compartments to protect them from nuclease digestion [34,37]. To explore the significance of this phenomenon for bkv-miR-B1-5p, we processed the conditioned medium from BKPyV-infected Vero cells through 100,000× *g* ultracentrifugation and then a sequential ultrafiltration procedure (Figure 4a,b). The supernatant from 100,000× *g* centrifugation of conditioned medium was filtrated through a 300 kDa MWCO membrane. The resulting filtrate was then filtrated through a 50 kDa MWCO membrane. No statistically significant change in the bkv-miR-B1-5p level was observed in the 300 kDa MWCO filtrate compared to the initial conditioned medium. In contrast, we highlighted a 2.76 ± 0.91 log10 copies/mL significant decrease of bkv-miR-B1-5p concentration in 50 kDa MWCO filtrate (*p* < 0.01). This suggests that most of the circulating bkv-miR-B1-5p is associated with proteins of molecular weight between 300 and 50 kDa, which is consistent with binding to Ago2. Hence, we performed immunoprecipitation for Ago2 and then quantified bkv-miR-B1-5p in anti-Ago2 and control immunoprecipitates and supernatants (Figure 4c). In comparison to controls (immunoprecipitation process without anti-Ago2 antibodies), the mean bkv-miR-B1-5p level was reduced by 30.3% in the supernatant (*p* < 0.05) and increased by 31.8% in the immunoprecipitate (*p* < 0.05). Overall, these results indicate that most of the extracellular bkv-miR-B1-5p is associated with proteins of molecular weight between 300 and 50 kDa, with an important proportion bound to Ago2.

### 2.5. bkv-miR-B1-5p to Distinguish Active and Persistent Infections

**Figure 5:**
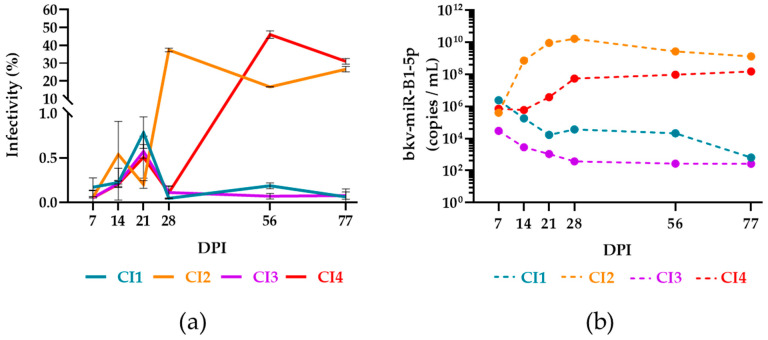
(**a**) Infectivity of conditioned media recovered from HRPTEC infected with 4 BKPyV clinical isolates (CI) 7 to 77 days post-infection (DPI). Infectivity rate corresponds to the percentage of cells that express BKPyV TAg among observed cells. The lines connect the mean infectivity rates between replicates. Errors bars represent standard deviation from the mean of duplicates. (**b**) bkv-miR-B1-5p level in conditioned media from HRPTEC infected with 4 BKPyV clinical isolates (CI) 7 to 77 days post-infection (DPI).

We have shown recently that the dynamic of bkv-miR-B1-5p level in urine from KTRs is different from the changes in BKPyV DNAuria load during chronic infection [28]. Viral DNA can be detected in the presence of defective viral genomes or neutralized virions [39]. Hence, it was supposed that bkv-miR-B1-5p, specifically transcribed during viral replication, would be a biomarker candidate to distinguish active and persistent infections. We thus aimed to develop a model to explore and confirm this phenomenon. Trying to reproduce the chronic infection in vivo, we screened 68 urine samples from KTRs, with highly detectable BKPyV DNA, to infect human renal proximal tubular epithelial cells (HRPTEC) based on a model for in vitro BKPyV propagation [40]. The cells were kept in culture for up to 77 days post-infection (DPI) with clinical isolates (CI). The culture medium was collected and changed every week. The efficiency of infection was monitored by qPCR for BKPyV DNA in the collected culture supernatant within the first four weeks of chronic culture. Four cell cultures were still positive for BKPyV DNA after 28 DPI and were kept for a longer time (77 DPI) while the others were stopped. We used the conditioned media collected at different time points of the 77-day-long chronic cultures to infect naïve HRPTEC. TAg immunostaining was performed at 7 DPI and the infectivity rate, representative of the active replication of BKPyV strains derived from CI, was measured (Figure 5a). TAg was significantly revealed only in cells infected with CI2 and CI4 supernatants. Moreover, the TAg expression was not observed with the infection by conditioned media collected before 28 DPI for CI2 and 56 DPI for CI4. To check if the infectivity of the samples was correlated with bkv-miR-B1-5p expression, we measured the concentration of bkv-miR-B1-5p in the conditioned medium from the four cultures chronically infected by CI (Figure 5b). For CI2 and CI4, the bkv-miR-B1-5p level was always higher than CI1 and CI3. The peak bkv-miR-B1-5p level for CI2 was observed at 28 DPI. It was higher and observed sooner than for CI4, the maximum of which was reached at 77 DPI. The kinetic of the bkv-miR-B1-5p level in conditioned medium was thus in line with the dynamic of the infectivity rate. The highest bkv-miR-B1-5p levels for CI1 and CI3 were obtained at 7 DPI and slightly decreased until 77 DPI. Accordingly, these results showed that high bkv-miR-B1-5p expression is associated with the presence of infectious BKPyV virions. Finally, bkv-miR-B1-5p turns out to be a relevant biomarker for predicting active BKPyV replication.

## 3. Discussion

In recent years, miRNAs have taken an important place in the perspective of new tools for diagnosis or prognosis of many pathologies. However, the direct application in clinical practice is still limited. This statement is particularly true for the management of viral diseases [41]. In fact, (RT-)PCR has become the gold standard for diagnosis and monitoring of most viral infections, especially for KTRs. Because of their lack of accuracy and more complex protocols, miRNA-targeting assays do not appear to be a relevant alternative to classical testing targeting viral DNA or RNA, but could be integrated as complementary viral biomarkers into a personalized medicine approach. We recently suggested that BKPyV miRNAs would be useful biomarkers to monitor the infection in KTRs, once the diagnosis is assessed by PCR. Emerging findings on other DNA viruses, such as *Herpesviridae*, confirm the potential of viral miRNAs to distinguish latent and active replication [41,42,43,44,45]. 

To verify this hypothesis on BKPyV, further evaluation in a large and multicentric cohort is necessary. The high stability of miRNAs in biological fluids, notably urine, has been commonly described [36] but specific knowledge of bkv-miR-B1-5p stability has never been studied. In the present study, we showed that bkv-miR-B1-5p is a suitable biomarker for retrospective studies, in vitro as well as in vivo. In both Vero and HEK-293TT cell BKPyV infection models, the extracellular bkv-miR-B1-5p level was not influenced by multiple freeze–thaw cycles (Figure 1a). We also observed a high stability of bkv-miR-B1-5p in cell lysate and supernatant from infected Vero cells, even after 80 days of room temperature storage (Figure 1b), as it has been described for other miRNAs [34]. The choice of the Vero cell line instead of HEK-293TT cells to evaluate long-term stability was motivated by a higher baseline bkv-miR-B1-5p level that could be more sensitive to observing significant decreases over time. The results may slightly differ between cell lines and could not perfectly reflect physiological concentrations. Moreover, we highlighted a non-significant decrease of the bkv-miR-B1-5p level in cell conditioned media after long-term room temperature storage. Turchinovitch et al. observed a significant diminution of several cellular miRNAs [34], but their analysis was based on relative, not absolute, quantities, which can affect statistical analysis and perception of the results [46]. In vivo, bkv-miR-B1-5p was still detectable after 5 freeze–thaw cycles in all tested urine (*n* = 5; Figure 2). However, this significantly affected the measured concentrations in two urine samples, highlighting the limit of such *a posteriori* study. We chose to test the condition for five, and not less, freeze–thaw cycles because of a lack of biological samples and to observe the more extreme conditions that would happen in laboratories. In fact, biological samples from biobanks are not often thawed many times before analysis. In order to evaluate extreme storage conditions and to compare the results to those observed in vitro, we conserved an aliquot for 80 days at room temperature. Overall, the bkv-miR-B1-5p level decreased in this condition, and the miRNA was undetectable in two urine samples. The biological and clinical characteristics of the five patients, summarized in Table S1, could not explain the observed variations. It must be noted that the remaining urines for patients #4 and #5 were conserved at +4 °C. Interestingly, the bkv-miR-B1-5p also decreased between day 0 and day 80 but remained detectable (Figure S1). Presence of RNAse, of which activity is inhibited at +4 °C, could explain this phenomenon. Moreover, the analyses of the different urine samples were independently performed over a year, while room temperature could differ for the distinct patients’ urine. Overall, the Mann–Whitney test did not reveal statistical differences between groups. To note, the Wilcoxon matched-pairs signed rank test was also applied and did not reveal significant differences between groups. Though, it was not relevant because the pairing between groups “Day 0” vs. “Day 80 at room temperature” was not significant (Spearman r = −0.56; *p* = 0.20). Actually, bkv-miR-B1-5p is highly stable in different matrixes as long as classical storage conditions are respected. Ideally, samples should be stored below −20 °C and not be thawed too many times.

Despite quantifying a significant amount, we found less than 1‰ of total extracellular bkv-miR-B1-5p in exosomes using two methods (Figure 3). This is consistent with previous studies showing that circulating cellular [34] or viral miRNAs [33] are marginally associated with exosomes. This confirms that bkv-miR-B1-5p can be quantified in the unprocessed extracellular milieu without the need for special isolation of exosomes. We showed that most of the circulating bkv-miR-B1-5p is associated with proteins of molecular weight ranging from 50 and 300 kDa (Figure 4a,b). This is consistent with the molecular weight of RNA-binding proteins (RBP), including Ago proteins, which are the major constituents of RISC to interact with miRNAs [37]. Indeed, according to our results, at least 30% of bkv-miR-B1-5p is associated with Ago2 (Figure 4c). This result may be underestimated because the immunoprecipitation method is not basically 100% efficient. Moreover, the remaining part of circulating bkv-miR-B1-5p could be associated with other Ago proteins (Ago1, Ago3, Ago4) [34,37] and/or it may be Ago-free in the miRNA-RBPs complex [47]. This mechanism must be explored in further studies to better understand the BKPyV life cycle, but the results would not affect the bkv-miR-B1-5p potential as a stable and abundant biomarker in the extracellular milieu. Unfortunately, we failed to obtain enough fresh, unprocessed, BKPyV-positive urine to perform these experiments on human samples. Exosomes and Ago2 are known to stabilize urinary miRNAs [48], but this remains to be demonstrated for bkv-miR-B1-5p. 

This study is the first one to explore the bkv-miR-B1-5p expression of HRPTEC, which were chronically infected with BKPyV CI. Browsing the literature, very few articles describe efficient methods for the in vitro propagation of BKPyV clinical isolates [40,49,50,51,52,53,54]. We empirically adapted a published method to develop our model of BKPyV CI screening [40]. The efficiency of the propagation seemed random, independently of clinical or biological characteristics of the patients at the time of urine sampling. Four CI allowed us to reproduce in vitro the chronic infection we observed in the previous clinical study [28]. In fact, two CI showed high and stable bkv-miR-B1-5p expression while the others showed lower and slightly decreasing levels. In our clinical retrospective study, we wondered if bkv-miR-B1-5p could be a better biomarker than BKPyV DNA to depict the intensity of viral infectivity in KTRs [28]. In the present study, based on our cell culture model, we confirmed that the circulating bkv-miR-B1-5p level was higher in samples that could infect other cells. Hence, the use of bkv-miR-B1-5p quantification in urine from BKPyV-infected KTRs would help the monitoring of infection. This could assist nephrologists to better anticipate the control of the infection and more rapidly adapt the therapeutic management, particularly the dosages of immunosuppressants, and limit the risk of immunological complications. However, the cut-off bkv-miR-B1-5p level to accurately distinguish active and persistent infection is still not defined and requires complementary studies.

## 4. Materials and Methods

### 4.1. Cell Cultures

The monkey-derived Vero cell line was obtained from the ATCC (ATCC-CCL-81). The human embryonic kidney HEK-293TT cells, stably transfected with pTIH plasmid containing the coding cDNA for SV40 TAg, were kindly provided by C.B. Buck. Both cell lines were cultured in Dulbecco’s modified Eagle’s medium (Invitrogen, Carlsbad, CA, USA) supplemented with 10% fetal bovine serum (Invitrogen). HRPTEC immortalized with pLXSN-hTERT retroviral transfection were obtained from Evercyte (Leberstraße, Austria; CHT-003-0002), maintained in a renal epithelial cell basal medium (REGM CC-3191, Lonza, Bale, Switzerland), and supplemented with human epidermal growth factor, 0.5% fetal bovine serum, hydrocortisone, epinephrine, insulin, triiodothyronine, transferrin, and GA-1000 (REGM SingleQuotsTM Supplement Pack, CC-4127, Lonza). All cells were cultured in a humidified environment with 5% CO2 at 37 °C. The protocol for the propagation of CI in HRPTEC was adapted from a previously described method [40]. Briefly, 5 × 10^4^ cells were seeded in each well of 24-well plate with 500 µL of medium. 100 µL of 0.22 µm filtered urine was used to infect the cell culture. The medium was removed and changed after 1 h of incubation. The cells were then kept for long-term culture, changing medium and collecting the supernatant every week.

### 4.2. Human Samples

Human urine samples were obtained from 73 KTRs followed at the Amiens University Medical Center (Amiens, France). The samples used for the present study were overages of the urine samples collected during routine patient management. 

Fresh urine samples were collected and aliquoted for conservation at room temperature, +4 °C, −20 °C, or −80 °C, depending on the experiments. The clinical and biological data of the five patients included in the stability experiments are summarized in Table S1. Sixty-eight urine samples from KTRs diagnosed with BKPyV DNAuria were randomly selected for chronic culture of BKPyV clinical isolates.

The study was conducted according to the guidelines of the Declaration of Helsinki and approved by the Ethics Committee of the Amiens University Medical Center (protocol code PI2021_843_0012, 15 December 2019).

### 4.3. Physico-Chemical Stability

Two million Vero or HEK-293TT cells were seeded in T75 flasks with 15 mL of medium and then infected with a Dunlop [38] BKPyV stock (MOI = 1), produced as previously described [55]. The supernatant was collected at 72 h, filtered at 0.22 µm and stored for 80 days either at room temperature or at −20 °C. The cell layer was lysed with radioimmunoprecipitate assay (RIPA)buffer (20 mM HEPES KOH pH 7.4; 150 mM NaCl; 5 mM EDTA; 0.1% sodium dodecyl sulfate (SDS); 1% deoxycholate (DOC) and 1% Triton X-100) and then diluted 1:10 in phosphate-buffered saline (PBS). The cell lysate was then filtered at 0.22 µm and stored for 80 days, either at room temperature or at −20 °C. 

Patient urine used in stability studies was stored at room temperature and at −20 °C for 80 days.

Aliquots were prepared at day 0 to have two separate aliquots at −20 °C to allow independent analysis of the effect of single freezing and 5 freeze–thaw cycles from −20 °C to room temperature. Freezing the 200 µL aliquots took approximately 1 h. Thawing the 200 µL aliquots took approximately 30 min.

### 4.4. miRNA Purification

Cellular and intravesicular miRNAs were extracted from centrifugation pellets or immunoprecipitates. These were lysed with 700 µL of Qiazol (Qiagen, Venlo, The Netherlands). Extraction was then performed using the “miRNEasy mini kit” (Qiagen) according to the supplier’s recommendations. Eluates were stored at −80 °C before analysis.

The miRNAs from culture supernatants, centrifugation supernatants or filtrates were extracted from 200 µL of the sample. The extraction was then performed using the miRNEasy Advanced Serum/Plasma Kit (Qiagen) according to the supplier’s recommendations.

Extractions from the different kits were performed on the Qiacube automated system (Qiagen).

Cel-miR-39-3p spike-in control, obtained from Qiagen, was added to the samples to either check the quality of the purification or normalize the bkv-miR-B1-5p qPCR results after successive filtrations and or centrifugations. 

### 4.5. miRNA RT-PCR Assay and BKPyV DNA qPCR

The miRNA RT-PCR assays and BKPyV DNA qPCR were performed as previously described [21,28].

### 4.6. Antibodies and Reagents

The mouse monoclonal anti-AgT antibody (pAb416) was obtained from Abcam (Cambridge, UK). For immunofluorescence experiments, the antibody (1 µg/mL) was diluted 1:1000. Anti-mouse IgG (H + L) antibody conjugated to the Alexa Fluor Plus 488 fluorophore was supplied by Thermofisher. For anti-Ago2 immunoprecipitations, the rat monoclonal antibody was provided by Millipore (clone 11A9, cat. MABE253).

### 4.7. Immunostaining

Cells were washed with PBS, fixed with 3.7% paraformaldehyde, and permeabilized with Triton X-100 (0.5% in cytoskeleton buffer: 10 mM PIPES (piperazine-N-N’-bis(2-etanesulfuric acid)), 300 mM sucrose, 100 mM NaCl, 3 mM MgCl_2_, and 1 mM egtazic acid). Infected cells were detected by TAg immunostaining. Nuclei were labeled by 4′,6-diamidino-2-phenylindole (DAPI). Immunostained cells were observed on a Zeiss Axio Vert.A1 microscope equipped with Colibri 7 LED 298 illumination (Zeiss, Oberkochen, Germany). Fluorescent signals were acquired with the Axiocam 305 (Zeiss). The percentage of infected cells was counted in an automated way using a modified version of the QuantIF macro [56].

### 4.8. Ultracentrifugation and Ultrafiltration

Two million Vero cells were seeded in T75 flasks with 15 mL of medium and then infected with BKPyV stock (MOI = 1). The supernatant was collected at 72 h post-infection and 8 × 10^9^ copies of cel-miR-39 were added to the supernatant to overcome volume variations related to subsequent manipulations and to allow normalization of results. The supernatant then underwent a succession of differential centrifugations to progressively remove various extracellular elements and purify exosomes according to a previously described protocol [34]: centrifugation at 1200× *g* for 3 min at room temperature to remove cells; centrifugation at 14,000× *g* for 10 min at room temperature to remove cell debris; filtration at 0.2 µm to remove microvesicles; ultracentrifugation at 110,000× *g* for 2 h at 4 °C to remove exosomes (the exosome-free supernatant was stored at 4 °C before further analysis); washing with PBS of the exosome pellet; and ultracentrifugation at 110,000× *g* for 2 h at 4 °C of the impure exosome–PBS mixture to remove traces of impurities. The ultracentrifugation supernatant was removed, and the pure exosome pellet was stored at −80 °C in Qiazol (Qiagen) for miRNA quantification.

Ultrafiltrations were performed from infected cell supernatants, centrifugation supernatants, or patient urine. The cartridges used for the filtrations were Vivaspin 20 with polyethersulfone membrane of pore size 10,000 MWCO, 50,000 MWCO, or 300,000 MWCO. Ultrafiltrations were performed according to the supplier’s recommendations. The retinates, parts retained by the column, corresponded to a 30X concentration of the starting solutions. Filtrates and retinates were stored at 4 °C if the secondary experiments were performed less than 24 h after ultrafiltration. Otherwise, the samples were stored at −80 °C.

### 4.9. Immunoprecipitation

Immunoprecipitation of extracellular miRNAs with anti-Ago2 antibody was performed using the µMACS™ Protein G MicroBeads kit from Miltenyi (Bergisch Gladbach, Germany) according to the supplier’s recommendations. The kit included beads, buffers, and columns. The treated samples were 30X ultrafiltration retentates of BKPyV-infected Vero cell culture supernatant. The maximum efficiency for immunoprecipitation was obtained for 50 µL of samples incubated with 2 µg of anti-Ago2 antibody. Immunoprecipitates were recovered by applying 700 µL of Qiazol directly to the column and miRNAs were extracted as previously described.

### 4.10. Statistical Analysis

Statistical analyses were performed using GraphPad Prism version 8.0.0 for Windows (GraphPad Software, San Diego, CA, USA). Nonparametric tests (Kruskal–Wallis test or Wilcoxon matched-pairs signed rank test) were performed. The threshold for statistical significance was set to *p* < 0.05.

## 5. Conclusions

Bkv-miR-B1-5p, a specific miRNA produced during active BKPyV replication, is a stable biomarker in samples from in vitro experiments and in clinical samples. Marginally associated with the exosomes, most of circulating bkv-miR-B1-5p is bound to proteins, especially Ago2, so the miRNA quantification does not require specific exosome isolation. Bkv-miR-B1-5p level is predictable of viral infectivity, which makes it a potential specific biomarker of active BKPyV replication in KTRs.

## Data Availability

The data presented in this study are available on request from the corresponding authors. The data are not publicly available due to ethical restrictions.

## Supplementary Materials

The following supporting information can be downloaded at: www.mdpi.com/article/10.3390/ijms23137240/s1.
