# Supplementary material for: BK Polyomavirus bkv-miR-B1-5p: A Stable Micro-RNA to Monitor Active Viral Replication after Kidney Transplantation
Source: Int J Mol Sci. 2022 Jun 29;23(13):7240. doi: 10.3390/ijms23137240 (PMC9266457; doi:10.3390/ijms23137240)
Supplement: Supplementary file 1 [file ijms-23-07240-s001.zip › ijms-1789402-supplementary.pdf]

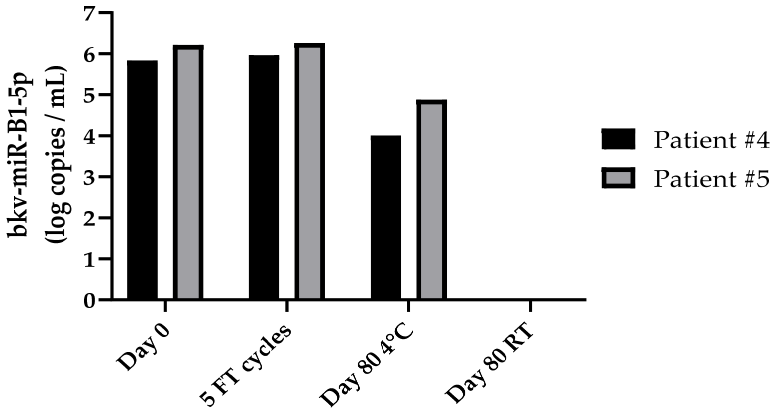

Figure S1. bkv-miR-B1-5p levels in urine from patients diagnosed for BKPyV infection. The measurements were performed at day 0, at day 80 after 5 freeze-thaw cycles, after 80 days of +4°C conservation, or after 80 days of room temperature conservation.

Patients	BKPyV DNA load (log10 UI/mL)	Days after diagnosis of BKPyV DNAuria	Days after diagnosis of BKPyV DNAemia	Creatinine (µmol/L)	Creatinine clearance (mL/min)
#1	Urine : 8,95<br>Plasma : 4,42	25	25	120	58
#2	Urine : 8,58<br>Plasma : NR	0	NR	66	84
#3	Urine : 7,1<br>Plasma : 3,5	188	112	241	26
#4	Urine : 9,1<br>Plasma : 5	141	94	359	15
#5	Urine : 9,1<br>Plasma : 4	150	0	125	53

Table S1. Biological and clinical information of the patients who provided the urines used for bkv-miR-B1-5p stability analysis. NR: not realized.
